# The Face of *Clostriodes Difficile* Infections in the Outpatient Setting

**DOI:** 10.51894/001c.12883

**Published:** 2020-06-08

**Authors:** Michael S Wang, Samad Faheem, Joanna Mangio, Kevin Pham, Daniel Lloyd, Brianna Hatch-Vallier, Ewanah Johnson

**Affiliations:** 1 Medicine Spectrum Health-Lakeland

**Keywords:** severity markers, ambulatory, clostriodes difficile infection

## Abstract

**BACKGROUND:**

It has long been well-established that Clostridiodes difficile infections (CDI) can cause severe morbidity and mortality. However, most of the literature to date has focused on hospital-diagnosed infections with less emphasis on clinic-based CDI cases. Guidelines from the 2010 IDSA/SHEA for CDI advocate for metronidazole as first-line therapy for mild to moderate CDI cases. However, the 2017 guidelines recommend oral vancomycin or fidaxomicin as first-line therapy due to their superior efficacy. Objective: The purpose of this study was to compare Clostriodes difficile infections in convenience samples of clinic vs. hospital patients.

**METHODS:**

In 2019, a retrospective, case-controlled study was performed by the first six authors between 2015-2017 (i.e., prior to the 2017 IDSA/SHEA CDI guidelines) to compare ambulatory and hospital CDI treatment prescriptions. Analytic data included frequency of White blood cells (WBC) and creatinine collection, frequency of severe CDI cases, compliance with the 2010 guidelines, CDI recurrence, and mortality.

**RESULTS:**

An eligible subgroup of N = 92 hospital patients at Spectrum Health Lakeland were more likely to have WBC (98.4% vs 32.6%, p<0.001) and creatinine (97.8 vs. 39.4, P < 0.001) drawn than 184 patients receiving clinic-based care. Hospital sampled patients were more likely to have severe CDI (46.7% vs 6.7%, p < 0.001). Mortality was less common in hospital patients (1.1% vs. 7.6%, p = 0.017) and the recurrence rates were similar. (21.2% inpatient vs. 28.3% outpatient, (p = 0.224).

**CONCLUSIONS:**

Based on these results, assessment of CDI severity remains limited in the ambulatory population due to the lack of severity markers. It is unclear if this is due to lack of available laboratory resources or difference in clinical presentation. Of those sample patients who have available markers of severity, patients receiving clinic-based diagnoses were less likely assessed to have severe CDI. Keywords: Cloistriodes difficile infection, ambulatory, severity markers

## INTRODUCTION

*Clostridiodes difficile* (CDI) infections have produced significant morbidity and mortality in the United States.^1,2^ Although much of the CDI literature to date has been focused on hospital patients, there is increasing concern over the burden of CDI in ambulatory settings.^1,2^ Risk factors for community-acquired CDI are similar to hospital acquired CDI, including antibiotics, particularly high-risk antibiotics (e.g., clindamycin, fluoroquinolones, cephalosporins, and beta-lactams/lactase inhibitors).^1^

In a study of 14,453 patients across 10 sites, only 24% of cases were diagnosed in the hospital setting.^2^ In addition, patients thought to have community-acquired CDI have often had prior exposures in emergency rooms, dialysis centers, and physicians’ offices^1,2^ Recurrent CDI is also common in both the health care (14%) and community acquired settings (21%).^2^

In terms of CDI treatment recommendations, the previously established treatments have included oral metronidazole 500 mg every 8 hours and oral vancomycin 125 mg four times per day.^3^ According to the 2010 Infectious Diseases Society/Society of Hospital Epidemiology of America (IDSA/SHEA) CDI guidelines, vancomycin was previously recommended for severe CDI over metronidazole, although metronidazole had been recommended for mild to moderate CDI.^3,4^ Oral fidaxomicin 200 mg twice daily has also been shown to be efficacious for CDI.^5^

The most recent 2017 IDSA/SHEA guidelines now also recommend oral fidaxomicin or vancomycin for mild to moderate CDI cases unless the supply of the two medicines is limited or unavailable.^4^ The definition of severe CDI defined in both CDI guidelines has focused primarily on leukocyte count (> 15 cells^3^/uL, normal 4.5-11.0) or creatinine > 1.5 x baseline (normal creatinine 0.5-1.0 mg/dL).^4,6^ Severity markers often influence CDI treatment options, specifically the decision of whether to use vancomycin, metronidazole, or fidaxomicin.^3,4,6^ There may be limitations of these severity markers in certain populations, particularly those with hematologic malignancies.^7^ Our study sought to examine the utility of CDI markers in an ambulatory population versus an inpatient population.

It remains unclear what proportion of clinic-based physicians have the readily available laboratory assays when assessing patients for CDI. It is also unclear to what extent providers adhere to guidelines as recommended by the IDSA/SHEA. The purpose of this case-control study was to examine diagnostic and treatment patterns of outpatient CDI.

## METHODS

This was a single-center, retrospective, case control study, comparing patients in an clinic-based patient settings vs. patients in a hospital setting. The *C Difficile* Polymerase Chain Reaction (PCR) database was reviewed from the microbiology records, dating from January 2015 to September 2017. This study was conducted during the period from February 2019-August 2019 at Spectrum Health-Lakeland, a 200-bed community-based hospital with both teaching and non-teaching patient services in St. Joseph, MI.

The study hospital has a Level 3 Trauma Center with adult intensive care, medical, oncology, post-surgical, orthopedic, neurology, cardiac, and pediatric units. The Spectrum Health-Lakeland Institutional Review Board approved the study prior to data collection.

Case patients were defined as patients ≥18 years with a positive *C. difficile* stool specimen collected in an ambulatory setting. Case patients were excluded from the analytic sample if there was no reported diarrheal illness (i.e., ≥ three watery stools in a 24-hour period) associated with the positive stool specimen or they could not be matched to a control within 90 days of the specimen collection date.^1^ Control patients were defined as CDI patients diagnosed in a hospital setting.

Emergency Department cases were defined as a hospital setting and included as part of the control group. Each case was matched to two controls by sex and age within 10 years and matched within six months of diagnosis of CDI.

Patients were also be excluded if they had a prior CDI diagnosis within the prior six months. Additional data measures included white blood cell count (WBC), creatinine, recent antibiotic use within the last 90 days, recent hospitalizations, Charlson Comorbidity Index (CCI),^8^ and treatment medications prescribed. The CCI is a method of measuring the prognostic impact of comorbid disease, which factors in disease processes such as heart disease, kidney disease, liver failure, diabetes, and malignancy.^8^

A healthcare associated CDI was defined as an infection in any patient who had an overnight stay in a health care facility (i.e., hospital or nursing home) during the prior 12 weeks.^1^ Other measured outcomes included mortality and recurrent CDI among surviving patients.

Statistical methods included the use of the chi-square test of association, Fisher's Exact Probability Test, and ANOVA. Computations were performed via the VassarStats computational software^9^ by the first author (MW).

## RESULTS

During the study period, a total of 439 ambulatory patients with CDI (case) and 208 hospital patients with CDI (control) were initially identified. Of these patients, 255 case patients and 116 control patients were excluded based on age or CDI recurrence. A total of 92 (33.3%) eligible case patients and 184 (66.7%) control patients were identified.

The mean age in the case group was 61.7 (SD = 17.9) and the mean age in the control group was 63.4 (SD = 16.4), p = 0.443. (Table 1) Case patients were less likely to have been on recent antibiotics than control patients (6.9% vs. 46.7, p < 0.001). Case patients were also less likely to have health care CDI than control patients (22.8% vs. 53.8%, p < 0.001).

Case patients were also less likely to have had both WBC (32.6% vs. 98.4%, p < 0.001) and creatinine (28.3% vs. 97.8%, p < 0.001) levels drawn. In addition, case patients were also less likely to have significant morbidity, based on composite CCI scores^7^ (1.8 [0-12] vs. 3.2 [0-12], P < 0.001). Although the mean WBC between the subgroups was not significantly different, case patients were less likely to have a WBC >15 cells^3^/uL (6.7% vs. 21.5%, p = 0.040) and creatinine 1.5x baseline (3.8% vs. 23.3%, p = 0.013).

The authors’ ability to assess severity of CDI was diminished in case patients as compared to controls. (32.6% vs. 98%, p < 0.001). Among patients in which CDI severity could be assessed (Table 2), cases were less likely to be have severe CDI (6.7% vs. 46.7%, p < 0.001). Case patients were more likely to be treated with oral metronidazole (72.3% vs 49.5%, p=0.001) and control patients were more likely to be treated with either oral vancomycin or fidaxomicin (54.9% vs 25.8%, p < 0.001).

Thirty-day mortality occurrences were higher for control patients (7.6% [14 of 184]) than case patients (1.1% [1 of 92], p = 0.017). After accounting for mortality, recurrence of CDI within 60 days was similar in case (28.3%) and control patients (21.2%, p = 0.224).

**Table:** Table 1 – Baseline Clinical Data of patients with CDI

	**Ambulatory patients (Case)**	**Hospital patients (Control)**	**P value**
Age	61.7+17.9	63.4+16.4	0.443
% Male	32/92 (34.8%)	34.8% (64/184)	1.000
Nap1 ^+^	12.0%	11.4%	0.520
Health Care Associated	22.8% (22/92)	53.8% (99/184)	**< 0.001**
Charlson Comorbidity Index	1.8+2.3	3.2+2.4	**< 0.001**
Verified antibiotics within last 90 days	46.7% (43/92)	69.0% (127/184)	**< 0.001**
Fluoroquinolones	14.1% (13/92)	21.2% (39/184)	0.192
Penicillins	8.7% (8/92)	3.8% (7/184)	0.156
Beta-lactamase inhibitors	7.6% (7/92)	16.3% (30/184)	0.060
Cephalosporins	14.1% (13/92)	39.7% (73/184)	**< 0.001**
Marolides	1.1% (1/92)	3.8% (7/184)	0.285
Clindamycin	1.1% (1/92)	4.3% (8/184)	0.280
Trimethoprim	2.2% (2/92)	4.9% (9/184)	0.346
Carbapenem	3.3% (3/92)	3.3% (6/184)	1.000
WBC drawn	28.3%(30/92)	98.4% (181/184)	**< 0.001**
Creatinine drawn	39.4% (26/92)	97.8% (180/184)	**< 0.001**

+ North American pulsed-field gel electrophoresis type 1 strain

**Table:** Table 2 – Clinical Data of Patients with CDI

	**Ambulatory patients (Case)**	**Hospital** **patients (Control)**	**P value**
**WBC**	**10.1+4.4**	**11.7+6.6**	**0.221**
%WBC>15 cells^3^/uL	6.7% (2/30)	21.5% (39/181)	**< 0.040**
Creatinine (mg/dL)	1.0+0.6	1.6+1.9	0.125
Creatinine > 1.5 x baseline	3.8% (1/26)	23.3% (42/180)	**< 0.013**
Albumin	3.5+0.6	3.4+0.7	0.538
Ability to determine severity	32.6% (30/92)	98.0% (182/184)	**< 0.001**
Severe C DIFF	6.7% (2/30)	46.7% (85/182)	**< 0.001**
Compliance with 2010 guidelines _6_	53.8% (14/26)	51.6% (94/182)	0.501
% treated with metronidazole	72.3% (48/66)	49.5% (91/184)	**< 0.001**
% treated with oral vancomycin or fidaxomicin	25.8% (17/66)	54.9% (101/184)	**< 0.001**
% non-compliance due to using vancomycin instead of metronidazole (non-severe)	83.3% (10/12)	37.5% (33/88)	**0.004**
Death within 30 days	1.1% (1/92)	7.6% (14/184)	**0.017**
Recurrence within 60 days	28.3% (26/92)	21.2% (36/170)	0.224

## DISCUSSION

To our knowledge, this is the first study to examine the assessment and treatment of severe CDI cases comparing only where patients were diagnosed (clinic patient vs. hospital patient). Previous studies involving outpatients have focused on location of exposure, specifically community-acquired vs. hospital-acquired CDI.^1,10^ Earlier studies have also found that there are clinical care exposures, including physician’s offices, physical therapist, hemodialysis, and outpatient surgery centers, not previously classified as health care associated CDI.^1^

As physicians in an emergency room setting presumably have access to hospital resources, emergency room cases were classified as hospital patients. In addition, hospital patients were more likely to have significant co-morbidities, based on the difference in their CCI^7^ scores. (p < 0.001) Not surprisingly, hospital patients were more likely to have health care-associated CDI than ambulatory patients, given their overall higher relative morbidity. (p = 0.017)

In our sample, outpatients were less likely to have either WBC or creatinine levels drawn. As a result, our ability to assess disease severity comparisons in our hospital sample patients was limited (p < 0.001), since CDI severity markers rely on such laboratory markers.^3,4,6^ This difference may be accounted for by either the differences in presentation or typical laboratory resources seen in outpatient settings. The higher severity of inpatients, may be due to the higher underlying morbidity within that population, as demonstrated by the difference in the CCI (p < 0.001), although the comparison may be limited due to the relative lack of outpatient CDI data.

Although our ability to assess severe CDI was limited in case (ambulatory) patients, severe CDI was rare. Depending on the availability of vancomycin and fidaxomicin, this also raises issues as to the optimal potential treatment regimens for CDI. For those clinic-based providers who opt to use metronidazole, this raises issues due to possibility of missing potential cases of severe CDI and treatment appropriateness.^3,4,6,11–13^

In this sample, physicians in the control group (hospital patient) were more likely to adhere to the 2010 CDI guidelines. (Table 2) However, this difference was likely accounted for mostly by a significant number of clinic-based physicians prescribing oral vancomycin instead of metronidazole, which is now consistent with first-line therapy according to the 2017 IDSA Guidelines.^4^

Metronidazole was previously listed as first-line therapy for mild to moderate CDI infections in the 2010 guidelines,^6^ but has been discontinued as first therapy in the 2017 guidelines.^4^ Although outcomes between metronidazole and vancomycin for milder CDI cases in another study have been initially found to be similar,^3^ a subsequent study demonstrated inferior outcomes of symptom resolution and recurrence for metronidazole.^11^

However, there are still providers who opt to use metronidazole for milder cases of CDI due to the significant cost of vancomycin and fidaxomicin.^3,12,14^ One recent study examined patients under 65 and found no significant differences between the use of vancomycin and metronidazole in milder cases.^13^ After factoring in patient mortality, our study demonstrated no differences mortality among surviving patients between outpatients and inpatients.^3,6,11^

This again raises the question of metronidazole prescriptions in the clinic setting, as these findings are not consistent with the 2017 CDI guidelines.^4^ As other modalities such as fecal transplants and bezlotoxomab, a monoclonal antibody, become increasingly become part of treatment regimens,^15–17^ the complexities of treating outpatients may only increase.

Strengths of this study include our case-control design, as we were able to generate a well-matched comparison group between hospital and ambulatory patients. In addition, our electronic health record (EHR) allowed us to obtain reliable data regarding concomitant results between stools and serum blood draws.

Although most providers at our institution used the same EHR, one study limitation was that there were a few providers who do not use the common EHR, perhaps influencing the availability of pharmaceutical data. In addition, many of the patients in the case group, specifically in the outpatient setting, did not have severity markers measured, but measuring the percentage of available markers was part of the study design.

## CONCLUSIONS

As clinic-based cases of CDI has been shown to impose a high burden,^1,10,18^ we have demonstrated some of the limitations in diagnosing and treating CDI in ambulatory patients. Future studies are required to examine diagnostic and treatment patterns and whether ambulatory providers adhere to updated CDI guidelines. Future studies could investigate the awareness of the new guidelines with respect to outpatient providers as compared to inpatient providers.

Based on these results, assessing CDI severity in the clinic settings remains challenging given the limited availability of laboratory diagnostics for clinic-based providers to check severity markers. Although our study demonstrated that severe CDI in outpatients is rare, there is still considerable debate iconcerning the role of metronidazole for CDI. Given the diagnostic challenges and frequent recurrences, clinic-based providers should exercise caution before prescribing metronidazole.

### Conflict of Interest

The authors declare no conflict of interest.
